# Narrative Medicine, Dementia, and Alzheimer’s Disease: A Scoping Review

**DOI:** 10.3390/healthcare13243321

**Published:** 2025-12-18

**Authors:** Venusia Covelli, Marina Angela Visco, Martino Feyles, Angelica Cristal Sirotich, Alessandra Marelli

**Affiliations:** 1 Department of Theoretical and Applied Sciences, e-Campus University, 22060 Novedrate, CO, Italy; marina.visco@uniecampus.it (M.A.V.); cristalsirotich@gmail.com (A.C.S.); alessandra.marelli@uniecampus.it (A.M.); 2 Department of Human and Social Sciences, e-Campus University, 22060 Novedrate, CO, Italy; martinomaria.feyles@uniecampus.it

**Keywords:** Alzheimer’s disease, dementia, narrative medicine, scoping review

## Abstract

**Background/Objectives**: In recent years, Narrative Medicine (NM) has gained prominence in the context of neurodegenerative diseases, such as dementia, offering tools to understand the subjective experience of illness and to improve the care relationship. **Methods**: This scoping review, conducted following the PRISMA guidelines, analyzed the scientific literature from PubMed, PsycInfo, Web of Science, and Medline, encompassing 10 contributions focused on NM and patients with dementia or Alzheimer’s disease. **Results**: The analysis identified three main themes: 1) narrative, memory, and personal identity, highlighting the role of narrative in preserving a sense of self; 2) personalization of care, oriented towards person-centeredness; 3) the use of narrative in a formative and reflective function as a tool to promote empathy, clinical awareness, and observation skills in the training of health professionals. **Conclusions**: NM confirms itself as a relational and reflexive paradigm, capable of humanizing care and promoting therapeutic pathways that are more inclusive and sensitive to the patient’s subjectivity.

## 1. Introduction

Narrative Medicine (NM) represents a clinical intervention methodology that considers narrative as a fundamental instrument for comprehending the multiplicity of perspectives involved in a patient’s illness event. This approach encompasses not only the patient’s experience and narrative but also those of caregivers (both formal and informal), healthcare professionals, and other individuals engaged in the person’s care. NM advances clinical practice through the development of narrative competence, which enables practitioners to recognize, interpret, and be moved by stories of illness, thereby supporting physicians, nurses, social workers, and therapists in enhancing the effectiveness of care pathways [1,2,3]. An appropriately collected illness story, including the personal dimensions of the subject, facilitates the formulation and implementation of a more effective, suitable, and collaborative care pathway. The primary objective of applying NM is to construct a personalized care narrative that fosters a therapeutic alliance between the patient (or their family or caregiver) and the healthcare team [4,5,6]. Narrating the illness experience accentuates critical aspects of the patient’s journey, not only as a recollection of the clinical process but also as a resource to enrich the affective, relational, and subjective domains. This methodology emphasizes the importance of communicative and ethical considerations, thereby advancing patient care and facilitating effective dialog with family members and caregivers [7,8]. Consequently, NM emerges as a promising tool for addressing ethical, relational, and care-related challenges associated with various pathologies, as well as for integrating the human dimension into care pathways. It is essential to distinguish NM from therapeutic narrative interventions. While narrative practices within clinical encounters may confer beneficial effects and support patients’ overall well-being, narrative therapy seeks explicitly therapeutic objectives aimed at alleviating psychological distress or mental disorders [9,10]. These objectives significantly diverge from those guiding the management of chronic or neurodegenerative conditions. Therefore, a clear distinction must be made: narrative therapy constitutes a psychotherapeutic intervention with direct clinical aims, whereas NM is a clinical methodology centered on listening, understanding, and integrating patients’ illness narratives into care. Any therapeutic advantages derived from NM should be regarded as secondary or indirect relative to its primary communicative and relational purposes.

Over the past two decades, NM has garnered increasing interest from both the national and international scientific communities, and substantial contributions to its effectiveness in addressing multiple clinical conditions are now documented [11,12,13,14,15,16]. Regarding the older adult population, NM has also demonstrated potential as a beneficial approach [17,18], particularly in the context of dementia and Alzheimer’s disease. Dementia is a chronic, degenerative condition frequently observed in older adults, characterized by a progressive and irreversible decline in functional capacity and autonomy, with significant implications for care and familial relationships [19]. Among various forms of dementia, Alzheimer’s disease is a specific neuropathological condition and represents the most prevalent cause of dementia. The different nosological subtypes of dementia extend beyond short-term memory impairment, involving a gradual and irreversible deterioration of primary cognitive and behavioral functions [20]. Dementia is commonly described as a ‘family’ disease, not solely because of a genetic component, but due to the profound impact it has on the relational dynamics and emotional systems within families [21]. In this context, in addition to pharmacological treatments, it is crucial to implement consistent, effective, and needs-centered communication strategies with family members, who often assume the role of caregivers early in the disease course and become key interlocutors in the care process for individuals with dementia [22].

However, to date, no literature reviews have summarized whether and to what extent NM has been applied in the context of dementia, particularly Alzheimer’s disease. Given the complexity and multidimensional impact of dementia, there is a strong need for methodological tools that can enhance communication, improve care quality, and facilitate the sharing of care pathways. Due to its potential to address the complexity of dementia care, NM emerges as a promising and meaningful approach. Accordingly, we conducted a scoping review with the following objectives: (1) to identify and analyze scientific articles dealing with the application of NM in the care settings of dementia and Alzheimer’s disease; (2) to analyze how NM is cited and used in the selected contributions, assessing its role, function, and impact in clinical and care practices.

## 2. Materials and Methods

### 2.1. Study Design

This scoping review was conducted in accordance with the PRISMA-ScR (Preferred Reporting Items for Systematic Reviews and Meta-Analyses extension for Scoping Reviews) guidelines. As this study is a review of previously published literature, ethical approval was not required.

### 2.2. Research Question and Conceptual Framework

The research question was structured using the PICo framework (Person, Intervention, Concepts, Context): Person (individuals with dementia and Alzheimer’s disease, their caregivers, and health professionals); Intervention (applications of NM approach or tools); Concepts (NM perspectives and co-construction of care narratives); Context (clinical–assistential settings for people with dementia and Alzheimer’s disease).

### 2.3. Search Strategy

The search was conducted in October 2024 across four databases: PubMed, PsycInfo, Web of Science, and MEDLINE. Search strategies were customized for each database by combining keywords related to ‘dementia’ (dementia OR Alzheimer’s OR cognitive impairment OR memory loss) with keywords related to ‘narrative medicine’ (narrative medicine OR narrative approach OR narrative-based medicine) (Table 1). No time limits were applied to the search, allowing for the inclusion of all relevant publications to date. The search targeted titles and abstracts. Filters were applied to include English-language publications and peer-reviewed or formally published academic sources. In PsycInfo, filters included peer-reviewed journals and the English language, while other publication types (e.g., articles, reviews, case studies, editorials, letters, conference abstracts, book chapters) were included through search strategy design rather than automatic database limits.

### 2.4. Eligibility Criteria

A wide range of publication types was considered to ensure a comprehensive overview, including qualitative and quantitative research articles, review articles, case studies, study protocols, brief reports, commentaries, letters, editorials, conference abstracts, posters, and book chapters. Gray literature was excluded because the review focused exclusively on peer-reviewed or formally published academic sources. Publications not available in English were excluded.

### 2.5. Study Selection

Two researchers independently screened abstracts and retrieved relevant full texts (VC; AM). If abstract information was insufficient, the full text was read. The quality of data extraction was checked by randomly selecting 20% of the abstracts for a second, blinded review by two other researchers (MAV; ACS). Each reviewer classified studies as excluded, eligible, or ambiguous. Eligible or ambiguous full texts were analyzed, with 10% double-checked independently by two reviewers. Discrepancies and disagreements were resolved by discussion. Results were synthesized narratively and supported by tables to map key characteristics and findings across included studies.

### 2.6. Data Extraction and Synthesis

Data were extracted using a structured form designed to capture key study characteristics and findings. Results were synthesized narratively and supported with tables that mapped methodological features, narrative components, and the primary results reported across included studies.

## 3. Results

**Figure 1:**
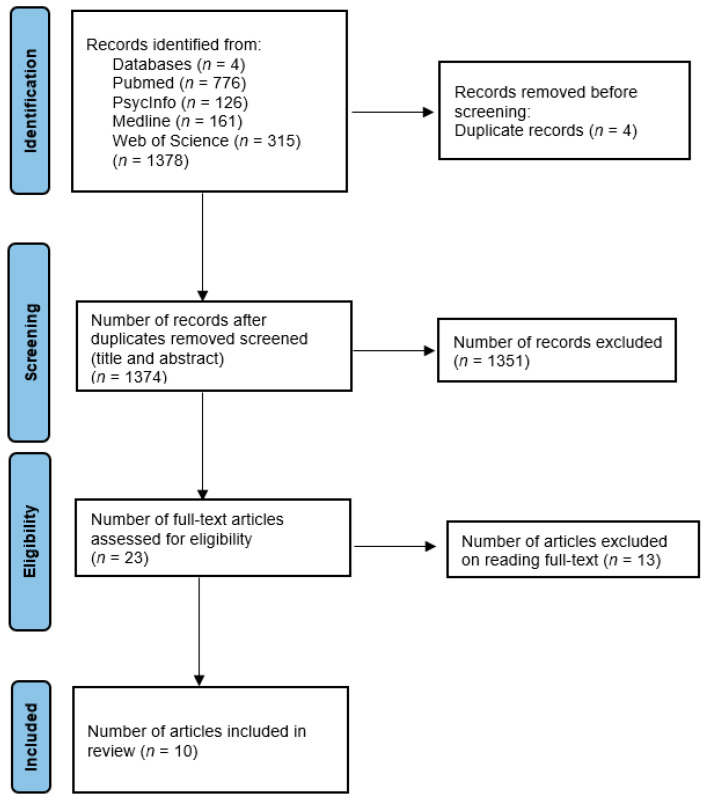
Flowchart of study selection.

After searching all four bibliometric databases, we identified 1378 records. After duplicates had been removed (N = 4), a total of 1374 records were screened, 23 full texts were read, and 10 studies were included, as shown in Figure 1. Studies were excluded if they were not available or did not address the topic of the present scoping review. A total of 1351 studies were excluded because the keyword “narrative” retrieved numerous literature reviews not directly related to this review’s focus. Additionally, many studies did not address the target population of people with dementia and Alzheimer’s disease. Furthermore, several excluded studies involved narrative approaches unrelated to NM, such as narrative therapy interventions. These exclusion criteria ensured that the review retained only studies specifically addressing the application of NM in the care of people living with dementia, thereby preserving its relevance and focus.

Table 2 delineates the principal characteristics of the studies included and summarizes the application of NM in care environments for individuals with dementia and Alzheimer’s disease. The reviewed literature predominantly originates from the United States [23,24,25,26,27,28], with additional contributions from Italy [29,30], Canada [31], and Poland [32]. The primary focus groups consist of individuals diagnosed with dementia or Alzheimer’s disease, alongside others involved in their care, such as caregivers, family members, and healthcare professionals. In certain instances, the focus also encompasses comorbid conditions such as breast cancer [23] or post-operative cognitive decline [25]. An illustrative example is the educational experience of a medical student engaged in home-based palliative care [24]. Participant data are not consistently available; however, two studies [26,30] report substantial sample sizes. Overall, the participant composition underscores the centrality of the person with dementia while also acknowledging the relational and institutional contexts in which care is administered.

Table 3 delineates the application of NM within the retrieved publications, emphasizing its purpose, employed instruments, and resulting outcomes. Several studies explicitly mention NM in their titles, abstracts, or keywords [23,26,30], whereas in other instances, the reference remains implicit or is confined to the introductory sections [27]. Lawrence [23] underscores the therapeutic benefits of group narratives, including the enhancement of empathy, the encouragement of introspection, and the promotion of personal development. Similarly, Cerasoli et al. [29] reported utilizing diaries as narrative tools to monitor neuropsychiatric symptoms in patients with dementia, actively involving caregivers in the process. An innovative approach, as described by Lingler et al. [26], involves the use of culturally adapted narratives aimed at increasing confidence and participation among African American adults in Alzheimer’s research. The study conducted by Puto et al. [32] highlights the role of storytelling in affirming the identity of older adults, thereby augmenting their sense of uniqueness and personal biography. Furthermore, Merrilees [27] integrates oral history techniques with personal narratives to foster dignity, empathy, and social inclusion. Joo, Li, and Whitlock [25] emphasize how the integration of narratives with clinical data facilitates a more profound understanding of postoperative cognitive decline, thereby overcoming the limitations associated with quantitative evidence alone. Tsai [24] offers a formative account that highlights clinical sensitivity towards patients with end-stage Alzheimer’s disease. Solomon and Lawlor [28] investigate ‘wandering’ in individuals with dementia through a phenomenological-narrative approach. Caza [31] underscores the benefits of autobiographical reminiscence. Lastly, Guidi [30] presents a particularly relevant application by illustrating the systematic incorporation of narratives into hospital communication protocols using the Calgary–Cambridge Guide, a tool designed to facilitate effective communication between healthcare professionals and patients.

## 4. Discussion

The analysis of the selected contributions confirms the growing interest in applying NM to dementia, particularly Alzheimer’s disease. Despite the heterogeneity of contexts, methodologies, and tools, it is possible to identify standard guidelines that enhance narrative as a clinical, educational, and relational resource. The first recurring area concerns the function of illness narrative collection in promoting the patient’s personal identity. In neurodegenerative conditions characterized by the progressive loss of cognitive functions, the possibility of telling one’s own story—or of seeing it said by others—represents a fundamental means of preserving the continuity of the self and the dignity of the individual [31,32]. Narrative practices, in this sense, are not limited to an expressive purpose but take on a therapeutic significance, insofar as they counteract processes of identity disintegration, often accentuated by stigmatization and social isolation [27]. A second interpretative axis is the personalization of care, a theme that is particularly evident in the contributions that apply narrative tools in care pathways. The diary proposed by Cerasoli et al. [29] is a practice that enables accurate assessment of neuropsychiatric symptoms, facilitates identification of triggering factors, and encourages caregivers to be actively involved in the patient’s daily management. In a similar vein, Guidi [30] systematically integrates illness narratives into clinical communication using the Calgary–Cambridge grid, facilitating the co-construction of meaning along the care pathway among caregivers, patients, and family members. The third thematic core concerns the use of narrative in a formative and reflective function, as demonstrated by Tsai’s [24] contribution, which recounts the experience of a medical student involved in a home visit to an Alzheimer’s patient in palliative care. Storytelling becomes a pedagogical tool to promote empathy, clinical awareness, and observation skills in the training of health professionals.

Furthermore, some studies highlight the potential of illness narrative collection to promote equity and inclusion in clinical research, as illustrated by Lingler et al. [26], who demonstrate that culturally adapted narratives can enhance the confidence and participation of African American individuals in Alzheimer’s studies. Such an approach proves crucial in a field like geriatrics, where minority groups are often underrepresented. Finally, contributions such as Lawrence’s [23] and Solomon & Lawlor’s [28] explore deeper, more philosophical dimensions of narrative, highlighting its capacity to foster interpersonal connections, process complex emotional experiences, and offer symbolic resistance to the fragmentation of experience caused by illness.

Despite the points of convergence, the studies differ in their areas of application, methodological tools, and cultural references. Merrilees et al. [27] and Puto et al. [32] valorize oral histories to promote dignity and reduce stigma, while other works, such as those by Tsai [24] and Joo, Li, and Whitlock [25], illustrate the integration of storytelling in home care or post-operative care settings. Finally, some studies explicitly address ethnic–cultural diversity in participation in care [26] and social biases in the management of behavioral symptoms [28].

Overall, the results show how NM, although still poorly systematized in the field of dementia, represents a versatile resource to enhance the quality of care, facilitate communication with caregivers, personalize patient biographies, and support caregivers in their clinical and human roles. However, further empirical research is needed to assess its effectiveness rigorously and to consolidate its integration into care protocols. Despite these promising findings, this review has some limitations. The selected contributions were heterogeneous, lacked formal criteria for assessing methodological quality, and varied in the explicitness of the narrative approach. Additionally, most studies were predominantly descriptive, preventing a systematic analysis of NM’s effectiveness in the examined clinical practices.

Future research should aim to clarify how NM might influence the care process, the health outcomes of adult patients with dementia or Alzheimer’s disease, the caregivers’ experience with care burdens, and communication dynamics among healthcare professionals, patients, and caregivers. There is a growing need for more methodologically rigorous research, such as controlled studies, longitudinal research, and mixed-methods approaches, to evaluate the effectiveness of narrative-based interventions across different stages of dementia and various care settings [33]. Further research should investigate, for example, how the various narrative tools discussed in this review can be incorporated into care pathways for these patients, assessing their feasibility and sustainability in real-world settings. The development of standardized narrative protocols could help incorporate NM into clinical guidelines and dementia care training programs.

## 5. Conclusions

This review emphasizes that NM offers an innovative, cross-disciplinary approach to dementia care, integrating clinical, relational, and cultural aspects. Despite varied application areas and methodological tools, all contributions acknowledge the importance of illness narratives in enhancing patient well-being, maintaining personal identity, and supporting more ethical, shared decision-making. Listening to individual stories helps reduce ineffective interventions and promotes personalized treatment plans. The ‘care story,’ co-created by patients, caregivers, and the team, serves not only as a clinical tool but also as a means to foster greater humanization, continuity of care, and alignment between expressed needs and therapeutic responses.

## Figures and Tables

**Table 1:** Database Search Strategy.

Database	Database-Specific Search Strings	Filters Applied:
PubMed	(“dementia” OR “Alzheimer*” OR “cognitive impairment” OR “memory loss”) AND (“narrative medicine” OR “narrative approach” OR “narrative-based medicine”)	Title/Abstract, English, Publication types: Research Articles, Reviews, Case Reports, Editorials, Letters, Conference Abstracts, Posters, up to October 2024
PsycInfo (via EBSCOhost)	TI (“dementia” OR “Alzheimer*” OR “cognitive impairment” OR “memory loss”) OR AB (“dementia” OR “Alzheimer*” OR “cognitive impairment” OR “memory loss”) AND TI (“narrative medicine” OR “narrative approach” OR “narrative-based medicine”) OR AB (“narrative medicine” OR “narrative approach” OR “narrative-based medicine”)	Filters: Peer-reviewed journals, English, up to October 2024
Web of Science	TS = (“dementia” OR “Alzheimer*” OR “cognitive impairment” OR “memory loss”) AND TS = (“narrative medicine” OR “narrative approach” OR “narrative-based medicine”)	Document Types (Article, Review, Proceedings Paper), Language: English, up to October 2024
MEDLINE (via Ovid)	(dementia OR Alzheimer* OR cognitive impairment OR memory loss) AND (narrative medicine OR narrative approach OR narrative-based medicine).	English, Publication types: Research Article, Review, Case Study, Editorial, Letter, Conference Abstract Date: up to October 2024

**Table 2:** Summary of Studies on Narrative Medicine in Dementia and Alzheimer’s Disease.

Author (References), Year	Country	Type of Publication	Participants	Target	Diagnosis
Lawrence (2016) [23]	U.S.A.	Communication	N/A	Women with breast cancer; Staff in nursing homes for people with dementia.	Dementia and breast cancer
Tsai (2019) [24]	U.S.A.	Research Article/ End of Life Vignettes	A third-year medical student	A female with Alzheimer’s disease	Alzheimer’s disease
Joo, Li & Whitlock (2023) [25]	U.S.A.	Review	N/A	People with dementia; People with post-operative cognitive decline.	Dementia
Cerasoli et al. (2019) [29]	Italy	Communication	N/A	People with dementia	Dementia
Lingler et al. (2022) [26]	U.S.A.	Research Article	500 Black or African American adults	People with Alzheimer’s disease	Alzheimer
Puto et al. (2022) [32]	Poland	Communication	N/A	People with Dementia	Dementia
Merrilees (2023) [27]	U.S.A.	Mini review	N/A	People with Dementia	Dementia
Caza (2013) [31]	Canada	Brief review	N/A	People with Dementia	Older adults and patients with dementia
Guidi (2019) [30]	Italy	Conference Proceedings	267 older adult patients (169 patients with dementia)	People with dementia	Dementia and other pathologies
Solomon & Lawlor (2018) [28]	U.S.A.	Chapter	N/A	People with dementia and autism spectrum disorder	Alzheimer’s disease

N/A: Not available.

**Table 3:** Narrative Medicine Approaches: Aims, Tools, and Outcomes in Dementia Care Studies.

Author (Reference), Year	Keywords	Citation of “Narrative Medicine”	Aims	Type of Intervention/Tools	Results/Issues	
Lawrence, L. S. (2016) [23]	Braille; cancer; groups; narrative medicine; texts	NM cited in the title of the article and keywords	To explore how the narrative method in group settings enhances emotional processing, interpersonal connections, and mutual understanding.	Narrative tools: writing, reading, and sharing personal stories.	1. Enhancement of Connection and Empathy; 2. Facilitation of Emotional Expression; 3. Impact on Self-Understanding (deeper introspection); 4. Therapeutic Benefits.	
Tsai, J. (2019) [24]	Palliative care, home hospice, student, narrative medicine, home visit, medical education, Alzheimer’s, end of life	NM cited in the keywords only	A third-year medical student recounts an experience during a home visit of hospice and palliative care workers to a patient with Alzheimer’s disease.	End of Life Vignettes/physician’s narrative	The medical student participates in a home visit and shares their observations and impressions of the interaction, the older adult woman’s behavior, and the symptom challenges associated with her Alzheimer’s disease.	
Joo, Li & Whitlock (2023) [25]	Cognition, surgery, postoperative neurocognitive disorder, dementia	NM cited in the abstract	To explore how anecdotal evidence, NM, primary cohort studies, epidemiological research, and dementia literature can be connected to understanding long-term postoperative cognitive decline.	Anecdotal	NM enhances clinical management of postoperative cognitive decline by addressing overlooked subjective experiences, aligning patient and physician perspectives, and supporting more effective, personalized care alongside traditional scientific approaches.	
Cerasoli et al. (2019) [29]	Neuropsychiatric symptoms, dementia, narrative medicine, diary, assessment tools.	NM cited in the keywords and in the text	Discuss the potential advantages and implications of the diary for the ecological assessment of neuropsychiatric symptoms (NPS) in subjects with dementia.	Diaries	The use of the diary: 1. enables a more accurate description of symptoms; 2. facilitates the identification of triggering factors; 3. supports a personalized approach to patient management; 4. promotes active involvement of caregivers; 5. it can be utilized in research to monitor the effectiveness of interventions.	
Lingler et al. (2022) [26]	Alzheimer’s disease, Health equity, Narrative medicine, Recruitment	NM cited in the keywords only	To promote diversity in Alzheimer’s research by examining how culturally tailored narratives can inspire trust and encourage participation among African American adults	A culturally tailored narrative approach is implemented through the use of specific narrative materials.	Culturally tailored narratives build trust, reduce perceived burdens, and highlight benefits, effectively increasing African American participation in Alzheimer’s research and improving inclusivity, especially among those with prior research experience.	
Puto et al. (2022) [32]	Narrative; older people; long-term care; narrative medicine; identity.	NM cited in the abstract only.	To explore the role of NM in older adult care, particularly for patients with dementia.	NM skills	Incorporating narrative into medical care enables older adults to share their life stories, recognize their identity and uniqueness, and foster personalized care. It emphasizes that identity development continues throughout life and that narrative is a vital form of self-expression.	
Merrilees (2023) [27]	Oral history, personal narrative, values, aging, dementia oral history, dementia	NM cited in the text (Introduction).	The article examines the use of personal narratives to enhance person-centered care, improve empathy, reduce stigma, and support dignity and inclusivity for individuals with dementia and their caregivers.		Narrative-based intervention centered on the use of oral history techniques and narrative approaches, including active listening and open-ended interviewing.	Integrating personal narratives and oral history into dementia care fosters empathy, dignity, and collaboration, enhances communication, reduces stigma, and strengthens person-centered approaches, promoting social justice in aging and dementia contexts.
Caza (2013) [31]	Autobiographical memory, aging, dementia, narrative medicine	NM cited in the keywords only.	Presentation of a brief review of empirical data regarding the efficiency of narrative methods in the well-being of healthy older adults and individuals with dementia		Different narrative methods (examination of life, reminiscence, and autobiography)	Different narrative methods, based on memory evocation, would promote psychological adaptation among healthy older adults and those with dementia.
Guidi (2019) [30]	Older adult, fragility, communication, narrative medicine	NM cited in the title and abstract.	Presenting the experience of applying NM through the Calgary Cambridge Guide (CCG) in a post-acute operating unit.		Narrative interviews are used at three stages: opening interview, periodic communication during hospitalization, discharge, or death	Results highlight the importance of integrating communication into treatment plans and the need to enhance healthcare workers’ communication skills.
Solomon & Lawlor (2018) [28]	Alzheimer’s disease; Autism spectrum disorder; Dementia	NM cited in the abstract.	Through the lens of narrative and existential phenomenology, ethnographic accounts and published narratives by individuals with Alzheimer’s Disease were examined to understand their experiences of ‘wandering.’		Narrative-based interviews.	Writing as a way of contesting and resisting the decline of memory appears very significant in understanding the experience of dementia and ‘wandering’.

## Data Availability

No new data were created or analyzed in this study. Data sharing is not applicable to this article.

## Abbreviation

The following abbreviation is used in this manuscript:

NM	Narrative Medicine
